# Knowledge, attitude and perception towards lower limb amputation amongst persons living with diabetes in rural South Africa: A qualitative study

**DOI:** 10.4102/phcfm.v14i1.3398

**Published:** 2022-09-30

**Authors:** Eyitayo O. Owolabi, Kathryn M. Chu

**Affiliations:** 1 Centre for Global Surgery, Department of Global Health, Faculty of Medicine and Health Sciences, Stellenbosch University, Cape Town, South Africa

**Keywords:** lower limb amputation, diabetes mellitus, knowledge, attitude, perception, South Africa

## Abstract

**Background:**

South Africa has a high prevalence of diabetes mellitus (DM), a leading risk factor for lower limb amputation (LLA). Lower limb amputation is associated with significant morbidity and mortality. Lower limb amputation incidence can be mitigated through prompt identification and treatment of individuals at risk and engagement in self-management practices. Also, when LLA is inevitable, outcomes or prognosis can be improved with timely surgery.

**Aim:**

This study explored the knowledge, attitude and perception of persons living with diabetes towards LLA and its prevention.

**Setting:**

Nqamakwe, a rural community in the Eastern Cape province of South Africa.

**Method:**

This was a descriptive, qualitative study involving persons living with DM, with and without LLA, and community leaders. Fifteen participants were recruited purposively and conveniently from a rural community in the Eastern Cape, South Africa. Data collection took place through semistructured interviews, in English and a local language, Xhosa. Interviews were transcribed and translated, and an inductive approach was used for thematic analysis.

**Results:**

A total of 15 individual interviews were conducted. Of those, 13 were persons with DM, five with LLA, including one with bilateral LLA. There was a gap in knowledge on foot self-examination as a measure of preventing LLA amongst persons with DM. The attitude of persons without LLA was mostly fearful and their fears centred around perioperative death, risk for contralateral amputation, loss of limb and independence. Consent to LLA procedure was a last resort and only when pain levels were unbearable. Family support and information on rehabilitation services and assistive devices also fostered consent to LLA surgery.

**Conclusion:**

There is a need for awareness creation and adequate health education for persons living with DM on LLA and its prevention measures, especially foot care practices. Also, health education programmes for persons living with DM in rural areas should address the various misperceptions of LLA to reduce delays.

**Contribution:**

The article revealed gaps in knowledge on LLA and its prevention among individuals living with diabetes as well as areas of concerns that may potentially delay acceptance when LLA is inevitable. Findings from our study may assist primary health care providers to determine important issues to be addressed during routine and pre-operative patient education.

## Background

Lower limb amputation (LLA) involves the partial or complete removal of a limb^1^ and is associated with significant morbidity and mortality.^2,3^ The public health impact on the economic, social and psychological well-being of persons with LLA and their family members or caregiver is enormous.^4,5,6^

In South Africa (SA), diabetes mellitus (DM) prevalence is increasing because of population ageing, demographic transition, urbanisation, obesity and unhealthy lifestyle choices.^7^ Diabetes mellitus is a leading indication for LLA.^8,9,10,11,12,13^ Vascular complications from DM lead to the restriction of blood flow to the body extremities and increase the risk for foot infections and the need for LLA.^14^ Lower limb amputation amongst persons with DM is associated with high risk for perioperative complications, prolonged length of stay in hospital, increased hospital costs and mortality.^15,16^ Reported 30-day LLA-associated mortality rate ranges from 12% to 16% whilst annual mortality rate ranges from 43% to 48%.^16,17^

The incidence of LLA can be mitigated through proper DM management, prompt identification and treatment of individuals at risk and patient self-management practices including foot care and examination.^18,19^ Also, when LLA is inevitable, outcomes or prognosis can be improved with timely surgery.^20^ However, LLA refusal is common amongst persons with DM.^21^ Once needed, delaying the decision to amputate can increase mortality by 2% daily.^20^ Understanding of the pathogenesis of LLA and its prevention measures may influence their self-management practices like foot care and examination.^22^ Likewise, understanding of the benefits of timely LLA and the impact of delayed LLA on prognosis may foster acceptance when needed. Thus, concerted efforts towards improving knowledge of persons living with DM on LLA prevention and reducing delays in seeking care and decision-making are imperative for reducing LLA burden and improving its outcomes.

However, there is a gap in the literature on knowledge, attitude and perception of LLA and its prevention amongst persons with DM and the reasons that underlie acceptance or refusal of LLA when needed. Previous studies on LLA in SA majorly focused on the burden, indications and outcomes.^12,13,16,23^ The only existing study that assessed LLA knowledge was a quantitative study conducted amongst patients who were about to be amputated or had just been amputated^12^ and was not focused on prevention. Qualitative data helps to better understand the phenomenon of interest. To the best of the authors’ knowledge, the only qualitative study on LLA that exists in SA only looked at the experiences of living with LLA.^24^ Information on knowledge, attitude and perception towards LLA prevention amongst persons living with DM may help to design appropriate interventions or community engagement programmes for LLA prevention. Therefore, this study explored the knowledge of LLA and its prevention, as well as the attitude and perception towards it amongst adults with DM in a rural community in SA.

## Methods

### Study design and setting

This study used an exploratory, descriptive qualitative approach using a semistructured interview method for data collection. The study took place in Nqamakwe, a rural community in the Eastern Cape (EC) province of SA. The EC province was created in 1994, and includes areas from the Xhosa homelands of the Transkei and Ciskei, as well as part of the Cape province. The EC province is one of the poorest provinces in SA.^25^ The prevalence of DM in EC, SA is high, whilst the rate of glycaemic control is low.^26,27^ Nqamakwe is a village in the Amathole district municipality in EC, SA. It has a total population of 1558 and a population density of 1100/km^2^. It is largely dominated by black people (94.7%) and the predominant language in the village is isiXhosa.^28^

### Study sample and sampling

Fifteen participants were recruited purposively from a rural community in EC, SA, following a purposive and convenient approach. The study sample comprised three groups. The first group were persons with diabetes without amputation, identified from within the community through a nonprofit organisation (NPO) involved with the care and support of persons with chronic illnesses and disabilities. Identified persons were approached, and those who consented to the study were interviewed. The second group were persons with diabetes who had already undergone amputation. This group were recruited from the surgery database of the tertiary hospital in EC province where most amputations are conducted. The hospital provided a list of 45 people who underwent diabetes-related amputations in 2021. Only five persons who were reachable via telephone call and were willing to participate were recruited into the study. The third group comprised of the coordinator of an NPO working with people with chronic illnesses and disabilities (including amputation) and one community chief. The authors interviewed this group to understand the barriers or challenges faced by persons with DM-related amputations in the community.

### Eligibility criteria

All participants were adults, 18 years and older, residing in the study area. Exclusion criteria included the presence of cognitive or mental impairments or other health conditions that could affect the ability to partake in the interview, as well as persons with LLA unrelated to DM.

### Data collection

Data on age, gender, monthly income, marital status and length of DM diagnosis were collected. Knowledge of LLA, association with DM, its prevention as well as perceptions, attitudes and concerns towards LLA were explored using semistructured interviews. For those who already underwent LLA, the authors sought to ascertain reasons for potential delays and factors that influenced their decision to finally accept the procedure.

Interviews were conducted by the first author with the assistance of a trained local researcher using an interview guide informed by recent literature^1,29^ and the study objectives. The interview guide was piloted on two people living with DM and refined, and these results were excluded from the final analysis. Interviews were audio-recorded, and field notes were taken. Interviews were conducted in the respondents’ preferred language of choice and each lasted between 40 min and 60 min.

### Data analysis

All recorded interviews were transcribed and translated into English. A thematic analysis using an inductive approach was done. To ensure the accuracy of transcription, transcripts of a selected number of interviews were compared with the original recording by another language professional. The transcribed data were imported into NVivo 12 for data coding. Before coding, transcripts were read for familiarity and then followed by a more reflexive and critical reading of the transcripts. Then coding was done by the primary author, whilst two of the transcripts were independently coded by another experienced qualitative researcher to ensure the credibility of the analysis. Following that, data were grouped into issues that were related to the study objectives. After the coding was completed, the codes were refined and grouped into themes. Each theme was explored, subthemes were created where possible and irrelevant issues were teased out. Verbatim quotes were used to support the themes that emerged.

### Ethical considerations

Ethics approval to conduct the study was obtained from Stellenbosch University Health Research Ethics Committee (ref. no. N20/10/107) and the EC Provincial Department of Health. Verbal and written informed consent was obtained from the study participants after providing detailed information about the study. The assistance of a translator was employed and the opportunity to ask questions or clarifications was provided to participants before, during and after the data collection. Also, participants were informed of their right to refuse to participate or drop out whenever they deemed fit. Lastly, anonymity and confidentiality were ensured throughout the study and report writing by de-identifying all transcripts and analysis.

## Findings

A total of 15 individual interviews were conducted. Of those, 13 were done with persons with DM and two with community representatives. Of the 13 people with DM, eight had no amputation, four had unilateral LLA and one person had bilateral LLA. There were nine women and four men in the DM cohorts with a median age of 65 years (interquartile range [IQR] 58–69) and a median DM diagnosis duration of eight years (IQR 6–20) (Table 1). The two community representatives were a man and a woman. Further details of the demographic characteristics of each of the participants are presented in Appendix 1.

**TABLE 1:** Demographic characteristics of persons with diabetes.

Characteristics	Frequency	IQR
**Variable**		
Median age year	65	58–69
Median diabetes diagnosis duration in years	9	6–20
**Sex**		
Female	9	-
Male	4	-
**Amputation status**		
None	8	-
Unilateral	4	-
Bilateral	1	-

IQR, interquartile range.

Generally, whilst the participants demonstrated fair knowledge of the required lifestyle modifications, their knowledge on the role of these changes in LLA prevention was limited. Also, participants’ knowledge of foot care and examination as prevention measures for LLA was suboptimal. It was also found that persons with DM were fearful and anxious to discuss LLA and their fear centred around their perceived impending deaths or fear of contralateral amputation. The findings of this study are discussed in detail below under five major themes: knowledge of LLA and its prevention, attitude and perception towards LLA, pathway to LLA, decision-making regarding LLA and living with LLA. A summary of the identified themes and subthemes during the data analysis is presented in Table 2.

**TABLE 2:** Summary of prominent study themes and codes.

Themes	Subthemes	Codes
Knowledge of LLA and its prevention	Diet, exercise and medication	-
	Foot care and examination	-
	Deficiencies in patient education	-
Attitude and perception towards LLA	Fear and anxiety	Fear of impending death
	-	Fear of contralateral amputation
	-	Fear of impending altered body image
	LLA saves life	-
Pathway to LLA	Initial rejection and delay	-
	Consent	-
Decision making regarding LLA	The role of the family	-
	Access to assistive device	-
Living with LLA	Impaired functional ability	Impact on activities of daily living
	-	Impact on accessing healthcare
	Improved quality of life	-
	Depression	-

LLA, lower limb amputation.

### Knowledge of lower limb amputation and its prevention

Possession of adequate knowledge by persons with DM is an important step towards engaging in self-management behaviours and prevention measures for DM-related complications. Some of the measures for preventing LLA include behavioural and lifestyle modifications such as dietary changes, physical activities, smoking cessation, stress reduction, glycaemic control and (very importantly) regular foot care and examination.

#### Diets, exercise and treatment compliance

It was found that the most common knowledge amongst the study participants related to dietary changes, physical activities and the importance of compliance with treatment regimen. Even so, participants’ knowledge of these was basic and many were unable to clearly illustrate the link between these lifestyle changes and LLA prevention. Whilst some were unsure if LLA is preventable, others, especially those who had already undergone LLA thought that LLA is not preventable. These participants believed that they were compliant with recommended lifestyle changes and yet had LLA and therefore felt that diets, physical activities and even treatment compliance do not play a role in LLA prevention:

‘I don’t think I could have prevented the amputation, because I was taking my diabetes medications and yet I was amputated. I was also doing exercise, because I was told at the hospital to exercise. I was also told about what foods to eat, like eating a lot of vegetables and brown rice, which I always try to comply with.’ (Participant 8, 72 years old, female)

#### Foot care and examination

An important step towards the prevention of LLA is regular foot care and examination. Foot care and examination helps to promptly identify and seek care for any foot disorder. The study, however, found that participants’ knowledge of these was suboptimal. Some participants verbalised not knowing that their feet should be examined and that they never had their feet examined:

‘No, I was never told about the importance of foot examination, and neither has any doctor ever checked my foot since nine years that I have had diabetes.’ (Participant 6, 69 years old, female)

Some, however, misunderstood foot examination as symptom-based management and only engaged in foot care when they experienced symptoms. Because they lacked adequate knowledge of the importance of foot care and LLA prevention, they did not seek appropriate care when they discovered abnormalities:

‘I don’t check my feet unless I feel some hotness, which I only deal with by taking off my shoes and walking around with my barefoot. I don’t have anything on my feet or legs; I don’t have a wound or anything. I am fine since I take my treatment [*medications*].’ (Participant 2, 55 years old, female)

On the other hand, some participants, especially those who had already undergone amputation, appeared more knowledgeable about foot care and examination and verbalised engaging in these activities. It is possible that they were exposed to detailed education following their amputation or pre-operatively and were taking conscious efforts in preventing contralateral amputation:

‘Yes, I do check my feet … I used to look at my feet, do some exercises and wash them regularly.’ (Participant 13, 68 years old, male)

#### Deficiencies in patient education

Although participants highlighted different LLA information sources such as family members, media and the community, they blamed the healthcare providers for the deficiencies in their knowledge. Primary healthcare providers, especially nurses, are the first point of entry into the health system, and they conduct most of the health education. Persons with DM strongly rely on the nurses for the relevant information regarding their condition. The study findings, however, demonstrated deficiencies in the health education provided, especially around LLA and its prevention. Nurses’ knowledge of specialised topics such as diets and foot examination is also limited and they may not be able to provide all the necessary health education. Also, the primary healthcare centres are usually very busy with limited staffing; thus, health education may not be prioritised and when done, may be very brief and lack necessary details. It is also possible that the health education is conducted as a once-off and not regularly reinforced; therefore, some had forgotten what they were told:

‘I don’t know how I can prevent having a wound or when I have it how I can prevent amputation. I don’t know. I have never been educated about that or maybe I missed those lessons, but I’ve never been told how to avoid having a wound or amputation.’ (Participant 1, 52 years old, female)

### Attitude and perception towards lower limb amputation

Participants had various perceptions of LLA, and their attitudes and behaviour towards it varied.

#### Fear and anxiety

Feelings of fear and anxiety were prominent amongst those without LLA, whilst those who had undergone LLA were less fearful, although they were also fearful about it before the surgery. Whilst it was expected that persons with DM would have some form of awareness about LLA and how to prevent it, it was found that many of the participants were afraid and reluctant to discuss it:

‘I am afraid of amputation. I do not think anything about it, and I do not like to think about it because I am afraid. Oh! I do not want amputation.’ (Participant 6, 59 years old, female)

Participants’ fears were a result of various reasons and perceptions. One of the major sources of fear for the participants was the potential impending death that follows LLA. Lower limb amputation-related mortality is well documented and participants were aware of this, either through stories heard or the death of a known person who underwent LLA. This informed participants’ perception of LLA as a precursor to death. Whilst this is true, timely LLA may also reduce mortality risk, but there is a lack of awareness about this amongst the participants:

‘There are a lot of amputated persons who pass away. Once I know that I need to be amputated, I would know that I am closer to dying.’ (Participant 7, 69 years old, female)

Some were afraid of the risk of contralateral amputation. Some participants indicated that those who undergo LLA are more likely to have a contralateral amputation, which increases mortality risk. Even though this is true, it may not be the case at all times if measures to prevent contralateral amputation are in place. This relates to the lack of knowledge of LLA prevention by the participants or their perception that LLA is not preventable:

‘What I also do not understand about diabetes is that when you get amputated on one limb, you also get amputated on the other limb. It is scary.’ (Participant 2, 55 years old, female)

Another source of concern was the impending altered body image. Some could not come to terms with the fact that they would have to live without a limb, and this was a major cause of fear:

‘When the doctor mentioned amputation, I was afraid. I was afraid of being amputated. I was afraid of my leg being cut off.’ (Participant 8, 72 years old, female)

#### Lower limb amputation saves lives

It is, however, interesting to know that whilst many had negative attitudes and perceptions towards LLA, some considered it a form of help rendered by the doctors. These people had some form of knowledge of indications for LLA and understood that LLA is a form of treatment for nonhealing wounds. Therefore, they perceived LLA as a way of saving lives. Some participants also believed that the doctors know best and that their decision should not be questioned. They stated that the surgeons will only make amputation decisions when there are no better options and it is usually in the interest of the patient:

‘I think when a person undergoes amputation, it is because the doctors have seen that the legs are too damaged … the doctor is doing what is best for the patient.’ (Participant 1, 52 years old, female)

### Pathway to lower limb amputation

Lower limb amputation is preceded by a series of emotions and activities. This theme describes the various phases people went through before finally undergoing the LLA.

#### Initial rejection and delay

The initial reaction to the news of the need to undergo LLA was mostly rejection, which can be ascribed to the already highlighted fears and concerns. This is an expected behaviour as people will likely go through the various stages of grief, which include denial, anger or hurt, bargaining and depression, before finally consenting. All these potential delays should be anticipated by healthcare providers and they must start informing potential candidates way ahead of the planned surgery date:

‘When I was informed that I needed to be amputated, I was very hurt, so much that I said to him [*the doctor*] that I was going to come back to him because when I visited him I did not think that I would hear such news.’ (Participant 10, 59 years old, male)

Those who were not amputated also shared similar behavioural intent:

‘If my condition gets to the point when I am asked to undergo amputation, I will refuse. I wouldn’t just agree. I would have to go home and think about it at first.’ (Participant 2, 55 years old, female)

#### Consent

Despite the fears and anxieties towards LLA, and even the initial rejection or delay, many eventually consented to the surgery because they were left with no choice. This is usually the last resort, especially when they experience persistent and unbearable pain:

‘After I had initially rejected LLA, I didn’t stay for long after I got back from the doctor [*before accepting*]. So I stayed for a week bearing the pains but on the second week when I couldn’t take the pains anymore, I decided to go [*for the LLA*].’ (Participant 9, 73 years old, female)

Other factors that influence consent for LLA are discussed under the next theme, described as decision making regarding LLA.

### Decision making regarding lower limb amputation

Lower limb amputation is a devastating and life-altering DM complication. Therefore, consenting to LLA may be a difficult decision to make. It is therefore important for healthcare professionals to understand the issues confronted by patients during the decision-making process in order to develop an effective counselling strategy. Here, the authors describe the factors that influenced participants’ LLA decision making.

#### The role of the family

Friends and family are important support network for people living with diabetes and they play a key role in the decision-making process. The study participants emphasised the important role their family played in their decision making. As indicated by those who had already undergone LLA, one of the reasons for the delay was the need to inform their family of the latest development in their health condition and the doctor’s recommendation. Therefore, when LLA is required, involving the family members early enough in patient education may reduce delay in decision making:

‘When the doctor informed me about the need for amputation, I told them that I cannot be amputated because I did not tell my family; they did not know about the amputation so I needed to come back to the hospital after informing them.’ (Participant 8, 72 years old, female)

The family was also key in facilitating consent to undergo LLA. The study participants illustrated how they had initially refused LLA and how they eventually consented to it because of the counsel and support received by their family members. Because some of the worries of these persons are sometimes around altered body image because of limb loss or fear of rejection, positive affirmations and encouragement from family members can facilitate their decision:

‘When I was informed of the need for amputation, I was very hurt, so much that I said to him [*the doctor*] that I was going to come back to him … but my family said if that is the case, that I should do it that I cannot sit with a foot like that [an infected foot].’ (Participant 10, 59 years old, male)

#### Access to assistive devices

A major concern for those undergoing LLA is the resultant loss of function and living a dependent life, which is worsened when people do not have access to assistive devices. Even though crutches and walking aids seem to be the most common assistive devices provided, participants continually emphasised how they so much desired being provided with a prosthesis. They believed prosthesis would further improve their independence. One of the study participants stated how the pre-operative promise of a prosthesis influenced her decision to consent to surgery:

‘They explained that they were going to cut my leg and after they finish, I would get an artificial leg, and I agreed when they told me about the artificial leg. This explanation was provided when I returned for the amputation and not the first time when I was told I needed to undergo amputation.’ (Participant 8, 72 years old, female)

The reality, however, is that most may not obtain this prosthesis or may not obtain it in time. This is a common challenge in SA and other countries. Therefore, it is uncertain if such promises should be made to persons needing to undergo LLA. Rather, efforts to maximise and master the use of more feasible assistive devices like crutches and wheelchairs may be more realistic.

### Living with lower limb amputation

Lower limb amputation can bring about significant changes which may affect the amputated person in various ways. Under this theme, the authors describe what living with LLA looked like for the study participants, challenges faced in their individual lives and in the community.

#### Impaired functional ability

An important change highlighted by the participants was the inability to independently perform some of their usual activities of daily living and the need to rely on family members, friends or helpers. This was more challenging for those who were only provided with walking aids or crutches because of fear of losing balance or tripping, compared to those who had wheelchairs. The study participants were rural dwellers, mostly retired and low-income earners; therefore, they relied on the government for the provision of wheelchairs, which sometimes takes a long time:

‘They [*healthcare providers*] took my measurements, saying they will call me when they are ready to give me a wheelchair, but they said I must be patient because it is going to take some time.’ (Participant 10, 59 years old, male)

Even for those who had a wheelchair, moving around without assistance can be challenging in rural areas because of the poor road infrastructure and the lack of structures such as wheelchair ramps. One of the community representatives highlighted this challenge:

‘People with amputation struggle to move around in this community. The roads here [*in the rural communities*] are not conducive for wheelchair use; they only use their crutches, which is very difficult.’ (Community representative 1, age unknown, female)

In addition, LLA impacted participants’ ability to access care. Community health centres are located far away. Persons with amputations are limited in their ability to visit healthcare facilities for their regular check-ups because they rely on others to be able to travel to the clinics. If there is no available help, some miss their appointment dates. One of the community representatives also emphasised this challenge and opined on one of the proposed solutions by the community to work with the local health facilities in assisting those affected with medication refills:

‘We [*the community NPO*] are planning to work together with our community hospital to bring people’s medication closer to them. We have trained workers that may help to deal with this.’ (Community representative 1, age unknown, female)

#### Improved quality of life

Although in the community, persons with LLA are seen differently and usually approached with sympathy, possibly because of the perception that they are disabled and that they need extra care, interestingly, persons with amputation had a more positive feeling towards LLA following the surgery. This may be ascribed to the significant pain relief experienced by these people and the subsequent improvement in their quality of life, so much that they wished that they had not delayed their surgery. This positive feeling impacted their confidence and put them in a position to enlighten others on the importance of timely LLA when recommended:

‘I say to the people that if you are advised to undergo amputation of the leg, please get it done, and after that, you will live a longer life; you will live a good life as I am living now. I feel better now; I can go to places I want. I think I can be a good counsellor to others.’ (Participant 13, 68 years old, male)

#### Depression

In contrast, certain people who undergo LLA may struggle to deal with the resultant changes in their life. For instance, one of the community representatives reported a case of suicide by a community member who underwent diabetes-related LLA. According to her, the person was a young man and she ascribed the suicide to the lack of support from the man’s immediate family and the community:

‘One of the boys in the village [*who underwent diabetes-related LLA*] overdosed himself. He killed himself because he couldn’t take the stress and the reaction from his girlfriend and family.’ (Community representative 1, age unknown, female)

The study found contrasting views on the perceived level of support by the community for people living with amputation. Both of the community representatives felt that the community were either judgemental and did not show enough support to those who were amputated, or they exaggerated their care and concerns in a way that made the amputated person uncomfortable:

‘People [*community members*] may ask those who are amputated why they did not pay attention to their diabetes, or maybe they did not use their medications and then ended up being amputated. Some even make persons with amputation a laughingstock.’ (Community representative 1, unknown age, male)

On the contrary, the study participants who were amputated felt that the community were supportive:

‘The community members are supportive. Some ladies come and spend the day with me and chat; they will tell me that they’ve come to spend the day with me; they would ask how I am feeling.’ (Participant 9, 73 years old, male)

## Discussion

This study highlights a gap in the level of knowledge of LLA prevention, especially around foot care and examination amongst the study participants. This finding corroborates the reports from previous quantitative studies on LLA knowledge amongst persons with DM in SA.^8,30^ Studies have also shown the role of education and knowledge of DM disease process and foot care on health-seeking behaviour and improved health advocacy.^22^ Knowledge of foot care and engaging in recommended foot examination reduce the risk for LLA.^22^ The South African guideline for the management of DM recommends an annual foot examination and a more frequent screening for those at risk of foot infections.^31^ The deficiencies in the knowledge of LLA prevention amongst persons living with DM in the present study setting as well as the low level of foot examination are concerning. This may delay prompt identification and management of foot disorders and may consequently lead to increased LLA burden if prompt actions are not taken. Therefore, there is a pressing need to prioritise adequate and continual education of DM patients on LLA and its prevention and to design various prevention strategies and screening programmes, including foot examination.

This study showed high levels of fear and anxiety toward LLA, especially amongst those without amputation. A study from Ghana reported similar findings.^32^ These fears can lead to a high LLA refusal rate, thereby delaying sepsis control and increasing the risk of death. Healthcare providers need to understand the patients’ concerns and address them accordingly. For instance, explaining the LLA procedure in more detail including information on perioperative pain control may allay patients’ fears around pain. Adequate pre-operative teaching has been shown to reduce surgical patients’ anxiety.^33^ Likewise, the role of peer support and education should be explored pre-operatively to reduce delays when LLA is required. Those who had undergone LLA and now have a positive attitude towards it can be engaged to discuss their experiences and the potential benefits with the pre-operative patients. Peer support and education have been shown to be beneficial for those undergoing or who have undergone amputation, as this helps to reduce anxiety and deal with the fear of the unknown.^34^

Another factor which prompted some participants to agree to undergo LLA was the information provided on the potential of obtaining a prosthesis post-LLA. Prostheses, however, are not guaranteed, given the various challenges that surround accessing prosthetic devices in EC and SA at large.^35^ Some of these challenges include an already long prosthesis waiting list and backlog because of lack and unavailability, which also negatively impact prosthesis use and function.^36^ There also exists a range of other clinical factors, such as a lack of prosthetists or orthotists, which delays patient assessment and fitting. In addition, individual factors such as residual limb condition, financial instability for follow-up and purchase of required materials and even environmental factors all impact prosthetic services.^23^ Therefore, healthcare providers must be mindful of the promises offered and may consider more feasible options based on the resources at their disposal and the financial condition of the people.

Post-amputation, persons with DM had a more positive perception of LLA than they did pre-operatively, mostly because of the pain relief after the procedure. Family support also fostered a positive attitude towards LLA. Family and friends are important support networks, and they play a crucial role for persons undergoing LLA, before the surgery and during the transition phase after the surgery.^34^ One of the concerns of persons undergoing amputation is fear of rejection or abandonment by family.^37^ Therefore, having a family member who declares support before and after the surgical procedure may go a long way in reducing anxieties and delays and in promoting positive coping strategies and adjustments. Thus, the involvement of family members in patient education pre- and post-operatively should be prioritised by healthcare providers. This also helps the family members to better understand and prepare for the expected support role required of them.

Lastly, there were diverse perceptions about LLA amongst persons with DM in this study. Lower limb amputation was commonly perceived as a precursor to death. This perception mostly stemmed from previous experiences with friends or relatives who passed away following LLA. A similar finding was found in Nigeria.^1^ This is true, given the high rate of LLA-related mortality amongst persons with DM. However, several factors contribute to this increased risk for mortality. Firstly, people who need LLA usually have had long-term poor glycaemic control and likely underlying cardiac dysfunction from their DM, which increases mortality risk. Likewise, immediate perioperative mortality following LLA is high amongst DM individuals because of anaesthetic risk.^38^ The mortality risk is further increased amongst those who delay LLA. Health education on the pathophysiology of LLA in relation to DM, the importance of glycaemic control and timely surgery on prognosis may help those who require LLA to make a timely and informed decision.

### Limitations and strengths

This study is limited by its single study site, making its findings possibly less applicable to the wider DM community in SA or other countries. The under-representation of men may have impacted the findings of this study. Also, interviews were conducted over the telephone rather than in person because of the coronavirus disease 2019 (COVID-19) pandemic’s social distancing requirements, possibly limiting the interpretation of nonverbal responses from the participants. Also, the COVID-19 restrictions during the data collection period and the limited functioning of the primary healthcare clinics limited access to a large number of persons with DM. Therefore, the authors had to rely on those known and identified by the community leaders. Lastly, an interview is an inexact method to determine knowledge; thus, the study findings should be interpreted with caution. Despite these limitations, the study presents useful data including diverse opinions from nonamputated and amputated DM individuals as well as community support groups.

## Conclusion

There was a low level of knowledge about the need for self-foot examination as a preventive measure for LLA amongst individuals with DM. For those without amputation, the attitude towards LLA was mostly negative and characterised by fear. Lower limb amputation was commonly misperceived as a death sentence, bad luck and punishment. Awareness creation and adequate health education for individuals with DM on LLA pathogenesis and the importance of foot care practices for LLA prevention are needed. This should be prioritised by the primary healthcare providers, who promote preventative healthcare, and should be done at regular intervals during the routine clinic visits. A detailed explanation by the surgical team to the person undergoing LLA on the procedure, rehabilitation process and available resources such as walking aids, prostheses and wheelchairs and family involvement could reduce delay when LLA is required. Lastly, the use of peer counsellor(s) to educate and support people who must undergo amputations may be more credible than health care staff trying to explain and will save time for overworked health care staff.

## Appendix

**Appendix 1:** Demographic characteristics of the study participants

ID	Sex	Age (years)	Average income (Rand)	Marital status	Duration of DM diagnosis (years)
Participant 1	Female	52	1600	Widowed	8
Participant 2	Female	55	0	Married	20
Participant 3	Female	58	1890	Married	6
Participant 4	Female	60	0	Widowed	22
Participant 5	Female	62	0	Married	6
Participant 6	Female	69	1800	Married	9
Participant 7	Female	69	1800	Married	7
Participant 8†	Female	72	1900	Widowed	24
Participant 9†	Female	73	1750	Widowed	6
Participant 10†	Male	59	0	Single	19
Participant 11	Male	64	1800	Married	1
Participant 12†	Male	65	1800	Married	14
Participant 13‡	Male	68	1600	Married	5
Participant 14§	Female	-	-	-	-
Participant 15§	Male	-	-	-	-

DM, diabetes mellitus.

† , Persons with unilateral amputation.

‡ , Person with bilateral amputation.

§ , Community representatives, no demographic characteristics obtained.

## References

[1] Enweluzo GO, Ogbeide OA, Akinbode OO. Attitude, perception, acceptance, and life after amputation as seen in Lagos University Teaching Hospital. J Med Trop. 2019;21(1):37–41. 10.4103/jomt.jomt_5_19

[2] Zhang Y, Lazzarini PA, McPhail SM, Van Netten JJ, Armstrong DG, Pacella RE. Global disability burdens of diabetes-related lower-extremity complications in 1990 and 2016. Diabetes Care. 2020;43(5):964–974. 10.2337/dc19-1614 32139380

[3] Moxey PW, Hofman D, Hinchliffe RJ, Jones K, Thompson MM, Holt PJE. Epidemiological study of lower limb amputation in England between 2003 and 2008. J Br Surg. 2010;97(9):1348–1353. 10.1002/bjs.7092 20632310

[4] Sahu A, Sagar R, Sarkar S, Sagar S. Psychological effects of amputation: A review of studies from India. Ind Psychiatry J. 2016;25(1):4–10. 10.4103/0972-6748.196041 28163401 PMC5248418

[5] Mapa-Tassou C, Katte J-C, Maadjhou CM, Mbanya JC. Economic impact of diabetes in Africa. Curr Diab Rep. 2019;19(2):5. 10.1007/s11892-019-1124-7 30680578

[6] Bommer C, Sagalova V, Heesemann E, et al. Global economic burden of diabetes in adults: Projections from 2015 to 2030. Diabetes Care. 2018;41(5):963–970. 10.2337/dc17-1962 29475843

[7] International Diabetes Federation. Diabetes in Africa [homepage on the Internet]. No date [updated 2022 Feb. 03; cited 2021 July 21]. Available from: https://www.idf.org/our-network/regions-members/africa/diabetes-in-africa.html.

[8] Godlwana L, Nadasan T, Puckree T. Global trends in incidence of lower limb amputation: A review of the literature. S Afr J Physiother. 2008;64(1):8–12. 10.4102/sajp.v64i1.93

[9] Moxey P, Gogalniceanu P, Hinchliffe RJ, et al. Lower extremity amputations – A review of global variability in incidence. Diabetic Med. 2011;28(10):1144–1153. 10.1111/j.1464-5491.2011.03279.x 21388445

[10] Gutacker N, Neumann A, Santosa F, Moysidis T, Kröger K. Amputations in PAD patients: Data from the German Federal Statistical Office. Vasc Med. 2010;15(1):9–14. 10.1177/1358863X09346658 19841025

[11] Manickum P, Ramklass SS, Madiba TE. A five-year audit of lower limb amputations below the knee and rehabilitation outcomes: The Durban experience. J Endocrinol Metab Diabetes S Afr. 2019;24(2):41–45. 10.1080/16089677.2018.1553378

[12] Khan MZ, Smith MTD, Bruce JL, Kong VY, Clarke DL. Evolving indications for lower limb amputations in South Africa offer opportunities for health system improvement. World J Surg. 2020;44(5):1436–1443. 10.1007/s00268-019-05361-9 31897692

[13] Dunbar GL, Hellenberg DA, Levitt NS. Diabetes mellitus and non-traumatic lower extremity amputations in four public sector hospitals in Cape Town, South Africa, during 2009 and 2010. S Afr Med J. 2015;105(12):1053–1056. 10.7196/SAMJ.2015.v105i12.9276 26792165

[14] Adler AI, Erqou S, Lima TAS, Robinson AHN. Association between glycated haemoglobin and the risk of lower extremity amputation in patients with diabetes mellitus – Review and meta-analysis. Diabetologia. 2010;53(5):840–849. 10.1007/s00125-009-1638-7 20127309

[15] Aljarrah Q, Bakkar S, Aleshawi A, et al. Analysis of the peri-operative cost of non-traumatic major lower extremity amputation in Jordan. CEOR. 2020;12:13. 10.2147/CEOR.S232779 PMC6966148 32021336

[16] De Klerk C, Du Plessis G, Fourie JJ, O’Neill A, Smit SJA, Joubert G. The eventual outcome of patients who had lower limb amputations due to peripheral vascular disease at Pelonomi Hospital, Bloemfontein. S Afr Fam Pract. 2017;59(1):1–4. 10.1080/20786190.2016.1248145

[17] Husein S. Long term mortality after lower extremity amputation in South Africa [unpublished dissertation]. Department of Surgery. Cape Town: University of Cape Town; 2019.

[18] Creager MA, Matsushita K, Arya S, et al. Reducing nontraumatic lower-extremity amputations by 20% by 2030: Time to get to our feet: A policy statement from the American Heart Association. Circulation. 2021;143(17):e875–e891. 10.1161/CIR.0000000000000967 33761757

[19] Hingorani A, LaMuraglia GM, Henke P, et al. The management of diabetic foot: A clinical practice guideline by the Society for Vascular Surgery in collaboration with the American Podiatric Medical Association and the Society for Vascular Medicine. J Vasc Surg. 2016;63(2):3S–21S. 10.1016/j.jvs.2015.10.003 26804367

[20] Moxey PW, Hofman D, Hinchliffe RJ, et al. Delay influences outcome after lower limb major amputation. Eur J Vasc Endovasc Surg. 2012;44(5):485–490. 10.1016/j.ejvs.2012.08.003 22967904

[21] Godwin Y. We need to talk about amputation – A difficult conversation in the developing world. PFMA J. 2020. [serial online]. [cited n.d.]. Available from: https://www.thepmfajournal.com/features/features/post/we-need-to-talk-about-amputation-a-difficult-conversation-in-the-developing-world.

[22] Al-Wahbi AM. Impact of a diabetic foot care education program on lower limb amputation rate. Vasc Health Risk Manag. 2010;6:923–934. 10.2147/VHRM.S13569 21057577 PMC2964945

[23] Ennion L, Manig S. Experiences of lower limb prosthetic users in a rural setting in the Mpumalanga Province, South Africa. Prosthet Orthot Int. 2019;43(2):170–179. 10.1177/0309364618792730 30112980

[24] Godlwana L, Stewart A. The impact of lower limb amputation on community reintegration of a population in Johannesburg: A qualitative perspective. S Afr J Physiother. 2013;69(4):48–54. 10.4102/sajp.v69i4.379

[25] Statistics South Africa. South African Statistics [document on the Internet]. 2011 [cited 2021 July 21]. Available from: http://www.statssa.gov.za/publications/SAStatistics/SAStatistics2011.pdf.

[26] Adeniyi OV, Yogeswaran P, Longo-Mbenza B, Ter Goon D, Ajayi AI. Cross-sectional study of patients with type 2 diabetes in OR Tambo district, South Africa. BMJ Open. 2016;6(7):e010875. 10.1136/bmjopen-2015-010875 PMC4986079 27473948

[27] Owolabi EO, Goon DT, Seekoe E, Adeniyi OV. Correlates of pre-diabetes and type 2 diabetes in Buffalo City Municipality, South Africa. Afr J Phys Act Health Sci. 2016;22(41):1019–1035.

[28] Wikipedia. Nqamakwe [homepage on the Internet]. No date [updated 2021; cited 2021 July 21]. Available from: https://en.wikipedia.org/wiki/Nqamakwe.

[29] Yongu WT, Kortor JN, Mue DD, Anhange ST, Musa T. Patients’ perception and attitude to surgical amputation in Makurdi, North Central Nigeria. Niger J Med. 2020;29(4):623–627. 10.4103/NJM.NJM_122_20

[30] Olotu B, Anderson F. Knowledge and attitude of patients undergoing lower extremity amputation at RK Khan Hospital, Chatsworth. S Afr J Surg. 2019;57(4):9–13. 10.17159/2078-5151/2019/v57n4a2920 31773933

[31] Webb D. 2017 SEMDSA diabetes management guidelines. S Afr J Diabetes Vasc Dis. 2018;15(1):37–40.

[32] Amoah VMK, Anokye R, Acheampong E, Dadson HR, Osei M, Nadutey A. The experiences of people with diabetes-related lower limb amputation at the Komfo Anokye Teaching Hospital (KATH) in Ghana. BMC Res Notes. 2018;11(1):66. 10.1186/s13104-018-3176-1 29361966 PMC5781296

[33] Kearney M, Jennrich MK, Lyons S, Robinson R, Berger B. Effects of preoperative education on patient outcomes after joint replacement surgery. Orthop Nurs. 2011;30(6):391–396. 10.1097/NOR.0b013e31823710ea 22124192

[34] Sarah F. Practical coping strategies to help amputees and their families [serial online]. 2022 [cited 2022 Apr 27]. Available from: https://www.limbs4life.org.au/news-events/news/practical-coping-strategies-to-help-amputees-and-their-families.

[35] Mduzana LL, Visagie S, Mji G. Suitability of ‘Guidelines for Screening of Prosthetic Candidates: Lower Limb’ for the Eastern Cape, South Africa: A qualitative study. S Afr J Physiother. 2018;74(1):1–9. 10.4102/sajp.v74i1.396 PMC6093117 30135914

[36] De Boer-Wilzing VG, Bolt A, Geertzen JH, Emmelot CH, Baars EC, Dijkstra PU. Variation in results of volume measurements of stumps of lower-limb amputees: A comparison of 4 methods. Arch Phys Med Rehab. 2011;92(6):941–946. 10.1016/j.apmr.2011.01.007 21621671

[37] Roșca AC, Baciu CC, Burtăverde V, Mateizer A. Psychological consequences in patients with amputation of a limb: An interpretative-phenomenological analysis. Front Psychol. 2021;12:1–11. 10.3389/fpsyg.2021.537493 PMC8189153 34122200

[38] Stern JR, Wong CK, Yerovinkina M, et al. A meta-analysis of long-term mortality and associated risk factors following lower extremity amputation. Ann Vasc Surg. 2017;42:322–327. 10.1016/j.avsg.2016.12.015 28389295

